# Contribution of Chitinase A’s *C*-Terminal Vacuolar Sorting Determinant to the Study of Soluble Protein Compartmentation

**DOI:** 10.3390/ijms150611030

**Published:** 2014-06-18

**Authors:** Egidio Stigliano, Gian-Pietro Di Sansebastiano, Jean-Marc Neuhaus

**Affiliations:** 1 Laboratory of Cell and Molecular Biology, University of Neuchâtel; Rue Emile-Argand 11, Neuchâtel CH-2000, Switzerland; E-Mail: egidio.stigliano@ibbr.cnr.it; 2 Institute of Biosciences and Bioresources, National Research Council of Italy (CNR–IBBR), Corso Calatafimi 414, Palermo 90129, Italy; 3 ALSIA (Lucan Agency of Innovation and Technological Development)—Metapontum Agrobios Research Center, S.S. jonica 106, km 448.2, Metaponto 75012, Italy; 4 DiSTeBA (Department of Biological and Environmental Sciences and Technologies), University of Salento, Campus ECOTEKNE, S.P. 6, Lecce-Monteroni, Lecce 73100, Italy; E-Mail: gp.disansebastiano@unisalento.it

**Keywords:** chitinase, GFPChi, vacuole sorting, endoplasmic-to-vacuole (ERV)-pathway

## Abstract

Plant chitinases have been studied for their importance in the defense of crop plants from pathogen attacks and for their peculiar vacuolar sorting determinants. A peculiarity of the sequence of many family 19 chitinases is the presence of a *C*-terminal extension that seems to be important for their correct recognition by the vacuole sorting machinery. The 7 amino acids long *C*-terminal vacuolar sorting determinant (CtVSD) of tobacco chitinase A is necessary and sufficient for the transport to the vacuole. This VSD shares no homology with other CtVSDs such as the phaseolin’s tetrapeptide AFVY (AlaPheValTyr) and it is also sorted by different mechanisms. While a receptor for this signal has not yet been convincingly identified, the research using the chitinase CtVSD has been very informative, leading to the observation of phenomena otherwise difficult to observe such as the presence of separate vacuoles in differentiating cells and the existence of a Golgi-independent route to the vacuole. Thanks to these new insights in the endoplasmic reticulum (ER)-to-vacuole transport, GFPChi (Green Fluorescent Protein carrying the chitinase A CtVSD) and other markers based on chitinase signals will continue to help the investigation of vacuolar biogenesis in plants.

## 1. Introduction

Over the past few decades, glycosyl hydrolases of families 18 and 19, commonly known as chitinases (EC 3.2.1.14), have been studied for their importance in the defense of crop plants from pathogen attacks and for their peculiar vacuolar sorting determinants. While family 18 chitinases are found in all organisms, family 19 chitinases are mostly found in plants, although they have been found in a few animals, fungi, protists and in many bacteria. Until January 2014, 3374 chitinase DNA sequences were present in the NCBI database while the Pfam database revealed for Viridiplantae 569 sequences from 111 species for family 18 and 1047 sequences from 204 species for family 19 chitinases. They catalyze the hydrolysis of internal β-1,4-bonds between monomers of *N*-acetylglucosamine of chitin, the major cell wall structural polysaccharide of many fungi. This activity can evidently contribute to the defenses against fungal pathogens.

Unfortunately, the chitinases in cultivated plants can also have negative effects on human health since they may trigger allergic responses. Chitinases are major allergens in several food plants like banana, avocado or apple, as well as in rubber. Like many allergens, they are resistant to proteolytic degradation in the acidic environment of the stomach. This resistance favors entry into the bloodstream triggering the allergic response. The use of heterologous chitinases to engineer pathogen-resistant crop plants requires that the proteins are suitably modified to be optimized for pathogen resistance as well as to be properly hydrolyzed in an acidic environment [1].

Full understanding of the post-translational modifications (PTMs) of the engineered transgenic product is necessary both for the development of plants over-expressing defense proteins, and for the increase of nutritional value. Glycosylation certainly possesses a considerable importance, being the most abundant PTM in plant cells [2] and the most frequent post-translational modification related to the allergenic potential. However chitinases are rarely glycosylated. The catalytic domain shows a lysozyme-like folding in which two glutamates, Glu67 (number in barley chitinase) located in the third helix and Glu89 located in a more variable loop-like structure, act as main reactive groups for the inverting acid-base catalysis [3]. Additionally, Glu203 and Arg215 are both coordinated with Glu67. Not surprisingly, a similar triplet is shared with glycosyl hydrolases of families 46 (chitosanases) and 17 (β-1,3-glucanases) [4].

Family 19 chitinases have been subdivided in seven classes depending on the presence of a chitin-binding domain and on the presence of four loops within the catalytic domain. X-ray structures of several plant class I and II chitinases indicate a compact fold of the catalytic domain where the *C*-terminus is extended along the surface in such a way that additional residues would stick out over the catalytic cleft [5,6]. It is thus to be expected that the *C*-terminal propeptides found in many vacuolar class I chitinases are easily accessible to the sorting machinery as well as easily removed by endo- or exo-peptidases.

## 2. Chitinase Sorting, a Hidden Path

The vacuolar sorting determinant (VSD) residing in the *C*-terminal 7 amino acids long propeptide of tobacco chitinase A is both necessary and sufficient for the transport to the vacuole [7]. Fusion of this *C*-terminal propeptide to enzymatic or fluorescent reporters (rat β-glucuronidase and Green Fluorescent Protein (GFP)) confirmed these early evidences [8,9]. Only few other *C*-terminal VSDs (CtVSD) have been demonstrated to be involved in vacuolar sorting: a barley lectin [10], a β-1,3-glucanase and an osmotin from tobacco [11,12,13], 2S albumin storage proteins from Brazil nut [14], pea [15] and bean phaseolin [16].

Contrary to ssVSDs (sequence-specific Vacuolar Sorting Determinants, as identified, e.g., in barley for aleurain or sweet potato for sporamin), no specific sequence motif is needed for vacuolar sorting and different amino-acid substitutions led to different efficiencies of delivery to the vacuole. The importance of the *C*-terminus for the sorting machinery is stressed by the fact that the *C*-terminal Met of chitinase A could be replaced by a Phe or a Lys or even deleted, but not replaced by a Gly [7]. Similarly, addition of 1 or 2 Gly to the barley lectin propeptide or *C*-terminal addition of an *N*-glycosylation site caused a total secretion of the lectin [11].

The tobacco chitinase VSD has been less widely used than another *C*-terminal targeting signal, the tetrapeptide AlaPheValTyr (AFVY) from bean phaseolin [16]. There is no homology between these two CtVSDs but they now appear also to be sorted by different mechanisms [17]. The research using the chitinase CtVSD has been very informative, leading to the observation of otherwise neglected phenomena, such as the possible formation of two ontogenically distinct vacuoles [18], the presence of separate vacuoles in differentiating cells [19] and the existence of a Golgi-independent route to vacuoles [17].

Vacuoles are multifunctional organelles showing a high plasticity according to physiological/environmental needs [20,21]. The coexistence of two functionally and morphologically distinct vacuoles in single plant cells was confirmed by several studies involving different species [22,23,24,25]. Distinct vacuoles with different contents can certainly answer to specific physiological needs [19,25]. There is still a debate whether they derive by fragmentation from a pre-existing unique vacuole or develop from different prevacuolar compartments [26,27,28,29]. In some cases, as in programmed cell death (PCD) during pathogen response, vacuoles can fragment and acquire new functions that require specific membrane characteristics [30]. As for distinct biogenesis pathways, de Marchis and co-workers [31] recently reviewed several Golgi-independent pathways involved in the trafficking of different types of vacuolar proteins.

A recent electron microscopy study of *Arabidopsis thaliana* root tips using trafficking inhibitors [32] supports a model in which the lytic vacuole tonoplast forms from membranes derived from the endoplasmic reticulum, forming (empty) provacuoles. Similar models for the *de novo* biogenesis of a lytic vacuole had already been suggested long ago [33,34]. The provacuoles fuse with the pre-existing vacuolar network. Soluble contents then fill the vacuoles by the classical route via the Golgi. This corresponds well with the pattern observed with mini-protoplasts regenerating their central vacuole [18], when using the fluorescent vacuolar marker AleuGFP targeted with the sequence-specific VSD (ssVSD) of barley aleurain, a marker for lytic vacuoles, but not to what could be seen with the fluorescent vacuolar reporter GFPChi targeted with the CtVSD of tobacco chitinase-A [8]. This study also revealed that the regenerating central vacuole responsible for cell turgor is generated mainly by the lytic vacuolar compartment, labeled by AleuGFP. In contrast, GFPChi labeled small peripheral vacuoles, which we called pre-vacuoles. These fused with the central vacuole in a second step, probably at the end of cell expansion. In transgenic *Arabidopsis* plants expressing either marker, it could be seen that in nascent and expanding root hairs the central vacuole was labeled by AleuGFP, while GFPChi was excluded, while in fully expanded root hairs the latter marker also made it to the central vacuole [19]. This observation was not confirmed by other research groups using other vacuolar markers but can now be better understood considering the recent data [17] showing that GFPChi is a marker of a Sar1-independent traffic to the central vacuole. In our opinion, the central vacuole initially formed in mini-protoplasts [18] was filled by the standard Sar1-dependent pathway only, while the Sar1-independent pathway formed peripheral pre-vacuoles, supporting the idea of ontogenically distinct vacuoles.

In *Arabidopsis* plants accumulating anthocyanins, Brefeldin A (BFA) treatment did not alter the trafficking of either anthocyanins or GFPChi while it affected the trafficking of AleuGFP [35]. Similarly, for the enzymatic marker rat β-glucuronidase fused to the chitinase CtVSD (RGUSChi) it was wortmannin and not BFA that completely impaired vacuolar targeting in protoplasts [9]. The drug BFA is often used to demonstrate the involvement of Golgi in vacuolar trafficking (for a review see [36]) but it is also acting on endosomal compartments and on recycling to the plasmalemma, causing the formation of BFA bodies [37]. Recent results in *Nicotiana* [17], indirectly confirmed in tomato [38], indeed suggest that GFPChi transits via an intermediate compartment independent from the *trans*-Golgi Network (TGN) but involved in the late events of BFA bodies formation.

Other studies have however convincingly argued that GFPChi follows the canonical pathway through the Golgi–TGN–PVC (PreVacuolar Compartment). The functional studies of AtVPS45 [39] and AtVTI12 [40] showed that these proteins influence GFPChi sorting but mutants do not fully impair its transport to the central vacuole leaving open the possibility of a close interaction between the two pathways. Both AtVPS45 and AtVTI12 are positive regulators for vacuolar trafficking of cargoes carrying a CtVSD, being responsible for fusion processes at the tonoplast: AtVPS45 is a member of Sec1/Munc18 family and forms with AtVTI12 SNARE a multimeric complex also including SYP41/SYP61. In a *vps45* null mutant, the vacuole forms small concentric multilayer compartments stressing the involvement of post-Golgi trafficking in provacuole release and/or provacuole fusion [41] and opening to future model re-interpretation. The authors did not exclude the interference of an unbalanced SNARE (soluble *N*-ethylmaleimide-sensitive factor associated protein receptor) complex, composed of VAMP727, SYP22, VTI11, and SYP51. In fact it is known that the absence of both SYP22 and VAMP727 lead to vacuole fragmentation [42]. Over-expression of AtSYP51 also specifically interferes with GFPChi sorting to the vacuole [43,44]. SYP51 belongs to a new functional class of SNAREs with interfering activity, the i-SNAREs [45]. The i-SNARE activity of SYP51 is not shared by other related SNAREs such as SYP61 [43] and may be related to vacuole separation. SYP51 was localized on compartments of a different nature [43], which are distinct from Golgi and differently associated with free TGN [46], multivesicular bodies (MVB) (colocalizing with TGN and PVC markers) or tonoplast. For the investigation and characterization of the endoplasmic reticulum (ER)-to-vacuole pathways it is essential to identify valuable markers, such as chitinase. Even so many years after the discovery of the latter’s vacuolar targeting, the thorough study of the related-marker GFPChi leaves many unanswered questions.

A main issue is that putative vacuolar sorting receptor(s) for CtVSD proteins have been identified, but their sorting mechanism remains unclear. The CtVSD are not specifically recognized by the well-characterized Vacuolar Sorting Receptors (VSRs), which have a strong affinity for ssVSD [47,48]. These receptors have a similar structure to other vacuolar or lysosomal sorting receptors in animals or yeast, with a luminal cargo-binding domain, a single transmembrane domain and a short cytosolic tail with sorting signals controlling their incorporation into coated vesicles [48]. In 2000 [49], a new class of putative receptors, the RMRs (Receptor-like Membrane RING-H2), was identified in *Arabidopsis* based on the homology of its PA (Protease Associated) domain, with the PA domain, which is a part of the cargo-binding luminal domain of VSRs. *In vitro* binding assays of the luminal domain of RMRs have demonstrated an affinity for CtVSDs and not for ssVSDs, supporting their role as vacuolar sorting receptors for CtVSDs involved in sorting to the storage vacuole [50,51,52]. Co-immunoprecipitation and *in vitro* binding assays of AtRMR1 with phaseolin also support an *in vivo* interaction between proteins with a CtVSD and some members of the RMR family [51] (For a review on RMRs, see [53]). GFPChi may be targeted to the vacuole in an aggregated form as already shown for phaseolin [54,55]. The localization of RMRs is not clear yet: they have been localized in a DIP-labeled organelle (Dark Intrinsic Protein [49]), in the vacuolar lumen [56], in a new type of prevacuolar compartment [52] or different isoforms in either ER or a prevacuolar compartment (Occhialini, Gouzerh and Neuhaus, manuscript in preparation). These different results may be due to the different experimental approaches or organisms used and the discrepancies remain to be resolved.

## 3. Conclusions

The unclear results about sorting of markers with the chitinase A CtVSD suggests the existence of two different pathways for the chitinase: a classical pathway passing via the Golgi and the TGN and an alternative pathway in which soluble cargoes exit the ER by another Sar1-independent mechanism, delivering cargoes to the vacuoles without passing through the Golgi [17]. This pathway seems particularly important in developing seeds and involves the formation of dense vesicles. Already in 1993 Hara-Nishimura and co-workers demonstrated the existence of such an alternative pathway for the accumulation of 2S albumin and 11S globulins in pumpkin seeds via dense precursor-accumulating compartment (PAC) vesicles [57]. Over the years the number of proteins seen to bypass the Golgi has increased, including also the calcineurin B-like proteins [58], γ-gliadins [59], α-globulins [60], legumin-1 [61]. In rice, the formation of two distinct types of protein storage vacuoles involves the formation of PAC-like vesicles and of a new prevacuolar compartment [52,60].

**Figure 1:**
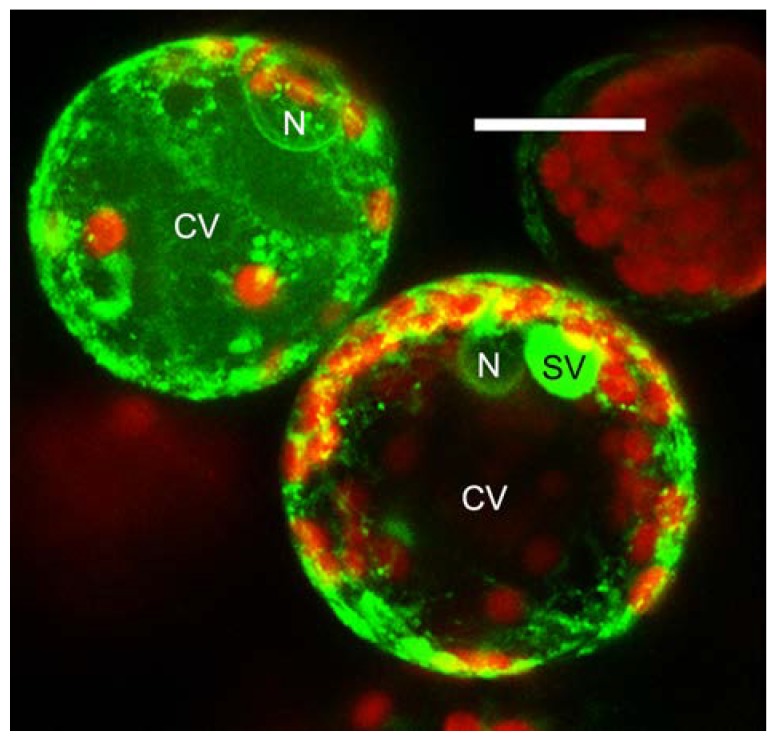
In this 10 μm projection of confocal images, two tobacco protoplasts over-expressing the Green Gluorescent Protein harboring the *C*-terminal Vacuolar Sorting Determinant of chitinase A (GFPChi, in green) accumulate the transgene following the two most described patterns. In the upper cell the transgene labels the endoplasmic reticulum (as the nuclear envelope is visible) but efficiently reaches the central vacuole. Several smaller compartments are also visible; In the lower cell the transgene also labels the ER but is not accumulated in the central vacuole. On the other hand, it is strongly accumulated in small vacuoles [8]. The red fluorescence of chlorophyll labels chloroplasts. N, nucleus; CV, central vacuole; SV, small vacuole. Scale bar = 20 μm.

*In vivo* observation of tobacco (Figure 1) or *Arabidopsis* protoplasts expressing GFPChi fluorescent shows that this marker is distributed among ER, punctate structures, and in either small vacuoles or in the central vacuole. In developing seeds, GFPChi accumulates in dense vesicles, while such vesicles are not observed in germinating cotyledons and GFPChi accumulates in ER bodies (Giselbert Hinz, unpublished observations). In vegetative tissues these dense vesicles do not seem to exist. How CtVSD proteins are sorted in these tissues remains to be determined. Combined with the use of new pathway selective inhibitors [62], GFPChi will continue to help dissect the mechanisms of vacuolar biogenesis in plants.
